# Simultaneous profiling of *Arabidopsis thaliana* and *Vibrio vulnificus* MO6-24/O transcriptomes by dual RNA-seq analysis

**DOI:** 10.1016/j.csbj.2021.04.008

**Published:** 2021-04-08

**Authors:** Yong-Soon Park, Jong-Seok Park, Soohyun Lee, Sung-Hee Jung, Seon-Kyu Kim, Choong-Min Ryu

**Affiliations:** a Biotechnology Research Institute, College of Natural Sciences, Chungbuk National University, Cheongju 28644, South Korea; b Department of Biological Sciences, College of Natural Sciences, Chungbuk National University, Cheongju 28644, South Korea; c Molecular Phytobacteriology Laboratory, Infection Disease Research Center, KRIBB, Daejeon 34141, South Korea; d Personalized Medical Genomics Research Center, KRIBB, Daejeon 34141, South Korea; e Biosystem and Bioengineering Program, University of Science and Technology (UST) KRIBB School, Daejeon 34141, South Korea

**Keywords:** Dual RNA-seq, Transcriptome, *Arabidopsis thaliana*, *Vibrio vulnificus*, *Plant-microbe interactions*

## Abstract

We previously demonstrated that a marine bacterial pathogen *Vibrio vulnificus* isolated from sea foods modulated gene expression levels and defense responses of a land plant *Arabidopsis thaliana*. Although the interaction between *V. vulnificus* and *A. thaliana* was verified under artificial and greenhouse conditions, the simultaneous changes in host and pathogen transcriptomes remained obscure. In this study, we simultaneously analyzed the transcriptome of *V. vulnificus* MO6-24/O and *A. thaliana* by dual RNA-sequencing analysis. Disease symptoms appeared at 5 and 7 days post-inoculation *in vitro* and post-infiltration *in planta*, respectively. A total of 31, 128, 303, 219, and 130 differentially expressed genes (DEGs) were identified in *V. vulnificus* MO6-24/O at 3, 6, 12, 24, and 48 h post-infiltration. Out of these, 14 genes involved in the virulence and pathogenicity of *V. vulnificus* MO6 were characterized. These genes were clustered into six categories, including adherence, antiphagocytosis, chemotaxis and motility, iron uptake, toxin and secretion system. In plant side, the bacterium DEGs potentially played a pivotal role in activating pattern recognition receptors (PRRs)-mediated defense responses. *A. thaliana* genes related to PRRs, reactive oxygen species burst, mitogen-activated protein kinase cascade induction, salicylic acid, jasmonic acid, ethylene, abscisic acid, auxin, gibberellin, and cytokinin were highly induced by *V. vulnificus* MO6-24/O challenge. Taken together, our results indicate that the sophisticated communication between a marine bacterial pathogen *V. vulnificus* and *A. thaliana* occurs. It is the first report demonstration that *V. vulnificus* actively modulates its virulence factors and potential host immune regulator in a land plant species.

## Graphical abstract

**Figure:**
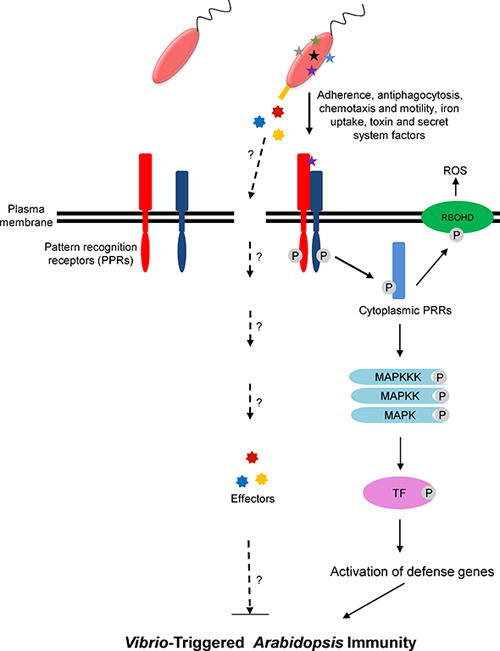

## 1 Introduction

*Vibrio vulnificus* is a Gram-negative bacterium belonging to the *Vibrionaceae* family and is found in warm coastal areas at temperatures ranging from 9 to 31 °C [1]. *Vibrio* species including *V. vulnificus*, *V. cholera*, and *V. parahemolyticus* causes severe diseases including cholera, gastroenteritis, eye and ear infection, and sepsis in human and livestock [2], [3]. Transcript profiles of five human pathogenic strains of *V. vulnificus* indicate that genes encoding the RtxA1 bacterial toxin, flagellar components, Flp-coding region, the GGDEF family protein, iron acquisition system proteins and sialic acid metabolic proteins play a role in the virulence of *V. vulnificus* [4]. In addition, RNA-seq analyses demonstrate that *Vibrio* species modulate the first layer of innate immunity of diverse hosts including fish and human, resulting in induction of toll-like receptors, RIG-like receptor, antioxidant genes, and NF-kB signaling genes [5], [6], [7]. Besides animal infection by *Vibrio* species, *V. vulnificus* also infects the land plant *Arabidopsis thaliana* under favorable conditions and regulates plant defense responses [8]. Although RNA-seq has been widely used to study the transcriptome of *Vibrio* species and its hosts including human, fish, and plants, the simultaneous spatiotemporal dynamics of the pathogen and host plant remains largely unknown.

Gene expression analyses can provide useful clues for understanding the molecular mechanisms underlying certain diseases and host–pathogen interactions, including *Vibrio*–plant interactions, and most of the new techniques used to quantify mRNA levels are based on polymerase chain reaction (PCR), first developed in 1984, such as quantitative PCR (qPCR), microarrays and RNA-sequencing (RNA-seq) [9], [10]. Among these methods, RNA-seq, which was developed more than a decade ago, has been used extensively to analyze gene expression under different conditions [10]. To date, RNA-seq analysis has been used to determine differential gene expression between different tissues in maize (*Zea mays*), *A. thaliana*, yeast (*Saccharomyces cerevisiae*), mouse (*Mus musculus*), and human (*Homo sapiens*) [11], [12], [13], [14], [15], [16], and to examine mRNA splicing and gene regulation by non-coding RNAs and enhancer RNAs [17], [18], [19], [20].

More recently, dual RNA-seq has been used to study gene expression and quantify mRNAs in both the host and its interacting pathogen simultaneously [21]. The first dual RNA-seq was performed in *Candida albicans* (animal pathogenic fungus) and mouse [22]. However, this study demonstrates closely related with computational model of host–pathogen interaction. To date, dual RNA-seq analysis has been used to study host–virus and host–parasites including bacteria and fungi interactions by high resolution techniques [23], [24], [25], [26], [27]. Although dual RNA-seq analysis of bacteria and their eukaryotic host is relatively difficult because the transcriptomic structure and numbers of prokaryotes is quite different from that of eukaryotes [21], dual RNA-seq is normally unique platform to understand microbial nature during host infection stages.

In this study, we performed dual RNA-seq analysis of *V. vulnificus* MO6-24/O (hereafter referred to as *Vv* MO6) during *A. thaliana* leaf infection. *In vitro* and *in planta* assays demonstrated that *Vv* MO6 caused disease symptoms in *A. thaliana*, and the population of *Vv* MO6 was 10–15-fold higher in *Vv* MO6 infiltrated *A. thaliana* leaf sample than in uninfected control plants [10% phosphate-buffered saline (PBS)]. The results of dual RNA-seq analysis revealed that *Vv* MO6 genes grouped in six categories, including adherence, antiphagocytosis, chemotaxis and motility, iron uptake, toxin, and secretion system. These genes were characterized for their potential ability to activate common pattern recognized receptors (PRRs) and plant immunity. In addition, plant genes involved in pathways mediated by defense hormones, including salicylic acid (SA), jasmonic acid (JA), and ethylene (ET), as well as abscisic acid (ABA)-, gibberellin (GA)-, auxin-, and cytokinin (CK)-dependent pathways were up-regulated at all tested time points compared with the negative control. Our results prove that gene expression profile of a marine pathogenic bacterium *Vv* MO6 and a land plant *A. thaliana* can be simultaneously analyzed from *Vv* MO6 infected *A. thaliana* under favorable conditions.

## 2 Materials and methods

### 2.1 Plant material and growth conditions

*A. thaliana* ecotype Columbia (Col-0) seeds were sterilized and germinated on half-strength of Murashige and Skoog (1/2 MS) medium, as described previously [8]. The germinated seeds were transferred to new growth media to prevent any contamination and incubated until needed for further experiments. The media composition and growth chamber conditions were the same as those reported previously [8].

To conduct *in planta* assays, Col-0, *fls2*, *efr1*, and *fls2/efr1* seeds were sown in soilless potting media (Punong, Gyeongju, South Korea), and seedlings were grown and incubated as described previously [8].

### 2.2 Analysis of disease severity and population density *in vitro*

Disease severity was examined as described previously [8]. Briefly, sterilized Col-0 seeds were grown on half-strength Murashige and Skoog (0.5x MS) medium supplemented with 3% sucrose and 0.6% plant agar and incubated in a growth chamber at 24 ± 2 °C under a 12 h light/12 h dark photoperiod for 3 days and germinated seeds were transferred to new medium for two weeks. Suspension cultures of bacterial pathogens, *Vv* MO6 and *Pseudomonas syringae* pv. tomato DC3000 (*Pto* DC3000; a positive control), were prepared, and their optical density was measured at 600 nm (OD_600_). Three to four leaves of Col-0 plants were drop-inoculated with *Vv* MO6 (OD_600_ = 2.0), *Pto* DC3000 (OD_600_ = 1.0) or 10% phosphate-buffered saline (PBS; a negative control) (Bioneer, Daejeon, South Korea). Disease severity was evaluated with ten plants per treatment at 5 days post-inoculation (dpi) [28]. The results were repeated at least 2–3 times.

To determine pathogen density *in vitro*, three to four leaves of 2-week-old Col-0 plants were drop-inoculated with *Vv* MO6 and *Pto* DC3000 for 5 dpi, harvested, homogenized, spread on LB agar medium containing 2% NaCl and 5 ug/ml rifampicin and LB agar medium containing 5 ug/ml rifampicin, respectively, and incubated at 30 °C for 2 days. Bacterial colonies were counted at 2 days after incubation. *Vv* MO6 and *Pto* DC3000 previously generated spontaneous resistance against rifampicin. The results were repeated at least 2–3 times.

### 2.3 Population density *in planta*

The population density of bacterial pathogens *in planta* was determined as described previously [8]. Briefly, suspensions of *Vv* MO6 (OD_600_ = 1.0) and *Pto* DC3000 (OD_600_ = 0.1) were infiltrated into the three to four leaves of 3-week-old Col-0, *fls2*, *efr1*, *fls2/efr1*, NahG, *sid2*, *npr1*, *jar1*, and *etr1* plants (n = 10), and bacterial cells were counted at 3, 4, and 7 dpi. The infiltrated leaves were homogenized, resuspended in 10% PBS, spread on LB agar medium containing 2% NaCl and 5 ug/ml rifampicin, and incubated at 30 °C for 2 day. The results were repeated at least 2–3 times.

### 2.4 Total RNA extraction, qRT-PCR, and RNA-seq library construction and massively parallel sequencing

Leaves of 3-week-old Col-0 plants were infiltrated with *Vv* MO6 suspension (OD_600_ = 1.0) and 10% PBS (negative control) and infiltrated leaves were harvested at 0, 3, 6, 12, 24, and 48 h post-infiltration (hpi). Three to four leaves were harvested in each plant and at least 20 plants were used for each treatment at each time point in two independent experiment. The harvested leaves were finely ground in liquid nitrogen using a mortar and pestle. Total RNA was extracted from approximately 100 mg of the powdered tissue using the RNeasy Plus Mini Kit, according to the manufacturer’s instructions (Qiagen, CA, USA). The quality of total RNA was analyzed by agarose gel electrophoresis and with a Nanodrop spectrophotometer (Nanodrop Technologies, Inc., DE, USA). First strand cDNA was synthesized using 2 ug total RNA, oligo-dT primer, random hexamer, dNTPs and Moloney murine leukaemia virus reverse transcriptase (M-MLV RT, enzynomics, Daejeon, South Korea. qRT-PCR was conducted using a Chromo4 Real-Time PCR System (Bio-RAD, CA, USA). Mixture contained 2x Brilliant SYBR Green Supermix (Bio-RAD, CA, USA), cDNA and 0.5 uM gene specific primer sets. The reactions were run using the following conditions: denaturation at 95 °C for 3 min, followed by 49 cycles of denaturation at 95 °C for 10 sec, annealing at 60 °C for 10 sec, and extension at 72 °C for 20 sec. Primer sets used in this study were listed in Supplementary Table 1.

The RNA-seq library was constructed using TruSeq Stranded Total RNA LT (with Ribo-Zero Plant) Set A (Illumina, CA, USA), according to the manufacturer’s instructions. The constructed dual RNA-seq library was sequenced on the Illumina HiSeq 2000 platform (Illumina, CA, USA) at the Korea Research Institute of Bioscience & Biotechnology (KRIBB), Daejeon, South Korea. The RNA-seq data were deposited in the NCBI Gene Expression Omnibus (GEO) public database under the accession number GSE159018.

### 2.5 Preprocessing and short read mapping

Illumina adapter sequences were removed from the sequence reads using cutadapt [29], and the preprocessing of reads was subsequently performed using DynamicTrim and LengthSort of the SolexaQA package [30]. The mean length of trimmed reads was 88.08 bp across all samples (minimum length = 25 bp). The trimmed reads were mapped to the reference genomes of *A. thaliana* (Araport) and *V. vulnificus* MO6-24/O (NCBI genome assembly accession no.: GCA_000186585.1) using the spliced-aware mapping algorithm implemented in HISAT2 (v2.1.0) [31]. Subsequently, the number of clean reads for each gene were counted using HTSeq (v. 0.11.0) [32]. To avoid bias due to differences in coverage depth among genes, the number of clean reads was normalized using the DESeq package in R [33].

### 2.6 Gene expression and hierarchical clustering analyses

Differentially expressed genes (DEGs) between samples were identified based on the fold change in gene expression and binomial test. False discovery rate (FDR), calculated using DESeq, was used as the threshold along with the *p*-value. Fold change was the ratio between normalized counts of treatment and normalized counts of control and *p* value adjusted for multiple testing with the Benjamini-Hochberg procedure. Correlation and hierarchical clustering analyses were performed using the AMAP library (version 0.8–14) and gplots (version 2.16.0).

### 2.7 Prediction of metabolic pathways

All DEGs were annotated by Gene Ontology (GO), [34], Kyoto Encyclopedia of Genes and Genomes (KEGG), and PATHVIEW analyses with Araport GO annotations. The number of GO terms and KEGG pathways in *A. thaliana* was assessed using in-house scripts (SEEDERS Inc., Daejeon, South Korea).

### 2.8 Statistical analysis

Analysis of variance (ANOVA) of the data was performed using the JMP software (version 5.0; SAS Institute, Cary, NC, USA). The significance of differences was determined based on the magnitude of the F-value (*P <* 0.05).

## 3 Results and discussion

### 3.1 *Vv* MO6 and *A. thaliana* interact both *in vivo* and *in vitro*

**Fig. 1:**
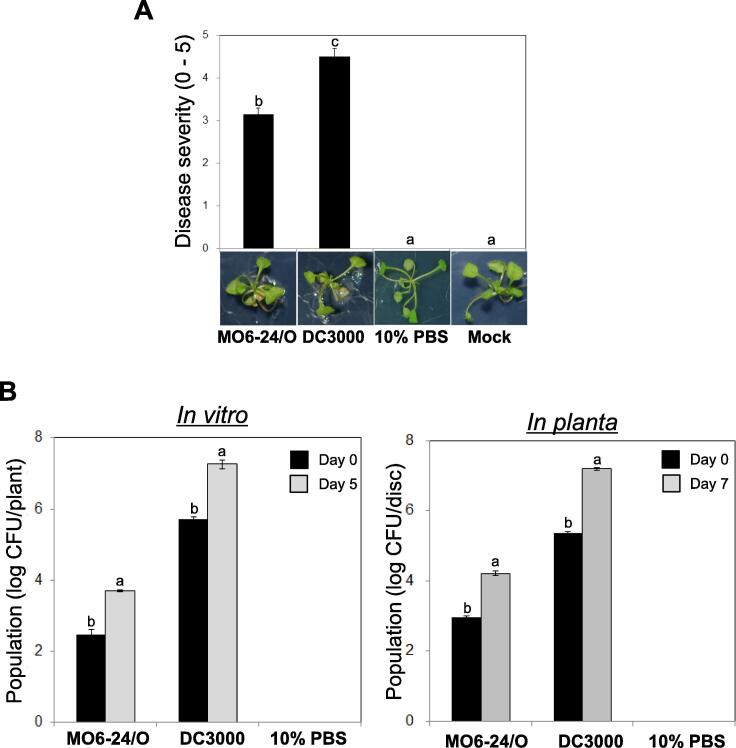
*Vibrio vulnificus* MO6-24/O (*Vv* MO6) and *A. thaliana* interact both *in vivo* and *in vitro.* (A) Assessment of disease severity in *A*. *thaliana* plants upon drop-inoculation with *Vv* MO6, *Pseudomonas syringae* pv. tomato (*Pto*) DC3000 (positive control) or 10% PBS (negative control). Photos were taken at 5 days post-inoculation (dpi), and disease symptoms were observed in by *Vv* MO6-inoculated plants. (B) Quantification of the population density of *Vv* MO6 in *A. thaliana* leaves. Approximately 15-fold higher population density was observed at 5 days after drop-inoculation *in vitro* and 7 days after infiltration *in planta*.

A marine bacterial pathogen, *V. vulnificus*, causes disease in the land plant *A. thaliana* under favorable conditions [8], suggesting that *V. vulnificus* affects expression levels of genes related with host defense responses. However, the mechanisms employed by *V. vulnificus* to modulate the host immunity remained unclear. In this study, we analyzed the transcriptomes of *V. vulnificus* and *A. thaliana* by dual RNA-seq analysis during their interaction. To optimize the conditions for host–pathogen interaction and to assess disease symptom in plants, leaves of *A. thaliana* plants were drop-inoculated with *Vv* MO6 or *Pto* DC3000 (positive control) *in vitro* and incubated for 5 days. After 5 days of incubation, plants inoculated with *Vv* MO6 and *Pto* DC3000 displayed disease symptoms, whereas uninoculated plants (mock) and plants inoculated with 10% PBS (negative control) showed no symptoms (Fig. 1A).

To confirm that the disease symptoms caused by *Vv* MO6 or *Pto* DC3000, we quantified the population density of the pathogens on leaves drop-inoculated *in vitro*. The inoculated leaves were harvested, homogenized and plated on LB agar medium supplemented with 2% NaCl and 50 µg/ml rifampicin (for *Vv* MO6-inoculated leaves) or with only 50 µg/ml rifampicin (for *Pto* DC3000-inoculated leaves). The population density of *Vv* MO6 and *Pto* DC3000 at 5 dpi was approximately 10- and 15-fold higher, respectively, than that at 0 dpi (Fig. 1B, left panel). To verify these results *in planta*, leaves of 3-week-old soil-grown *A. thaliana* plants were infiltrated with the *Vv* MO6 suspension, and the population density of *Vv* MO6 was quantified with culturable bacterium number on the media (Fig. 1B, right panel). The results obtained were consistent with the *in vitro* assay (Fig. 1B, right panel). Taken together, our results suggest that *Vv* MO6 causes disease in *A. thaliana* under tested conditions.

In fact, *Vv* MO6 originated from a patient who demonstrated seafood contamination-associated illness, whereas *V. vulnificus* 96-11-17M was obtained from the environmental sample [35]. The virulence and pathogenicity levels were similar between *Vv* MO6 and *V. vulnificus* 96-11-17M (data not shown). We hypothesized that a human pathogenic strain *Vv* MO6 well-adapted to host compared to an environmental strain *V. vulnificus* 96-11-17M. Therefore, we chose and tested *Vv* MO6 in this study. Thus, regardless of the origin, *V. vulnificus* strains cause disease in *A. thaliana*. This suggests that the virulence and pathogenicity of *Vibrio* species may be dependent on the conditions of the inoculated or infiltrated plant host. This is supported by the accumulating evidence showing that virulence and pathogenicity of *V. vulnificus* depend on the acidification, salinity, and carbon dioxide (CO_2_) levels in the host [36], [37], [38].

### 3.2 Gene expression profiles of *Vv* MO6 in *A. thaliana*

To demonstrate that *Vv* MO6 modulates host immunity, we investigated gene expression levels of *Vv* MO6 in *A. thaliana* leaves at 0, 3, 6, 12, 24 and 48 h post-infiltration (hpi). The expression levels of *Vv* MO6 genes 3, 6, 12, 24 and 48 hpi were normalized relative to those at the 0 h time point. A total of 4,470 *Vv* MO6 genes were analyzed by RNA-seq, of which 4,344 were functionally annotated. The trimmed reads, which get rid of *A. thaliana* sequences, were mapped on to the *Vv* MO6 reference genome (https://www.ncbi.nlm.nih.gov/genome/189?genome_assembly_id=165751), with an average mapping rate of 7.8% across six time points and 12 samples. The correlation coefficients of *Vv* MO6-infiltrated replicates were very high (range: 0.92–0.97), suggesting that no variation was present among replicates (Supplementary Fig. 1).

**Fig. 2:**
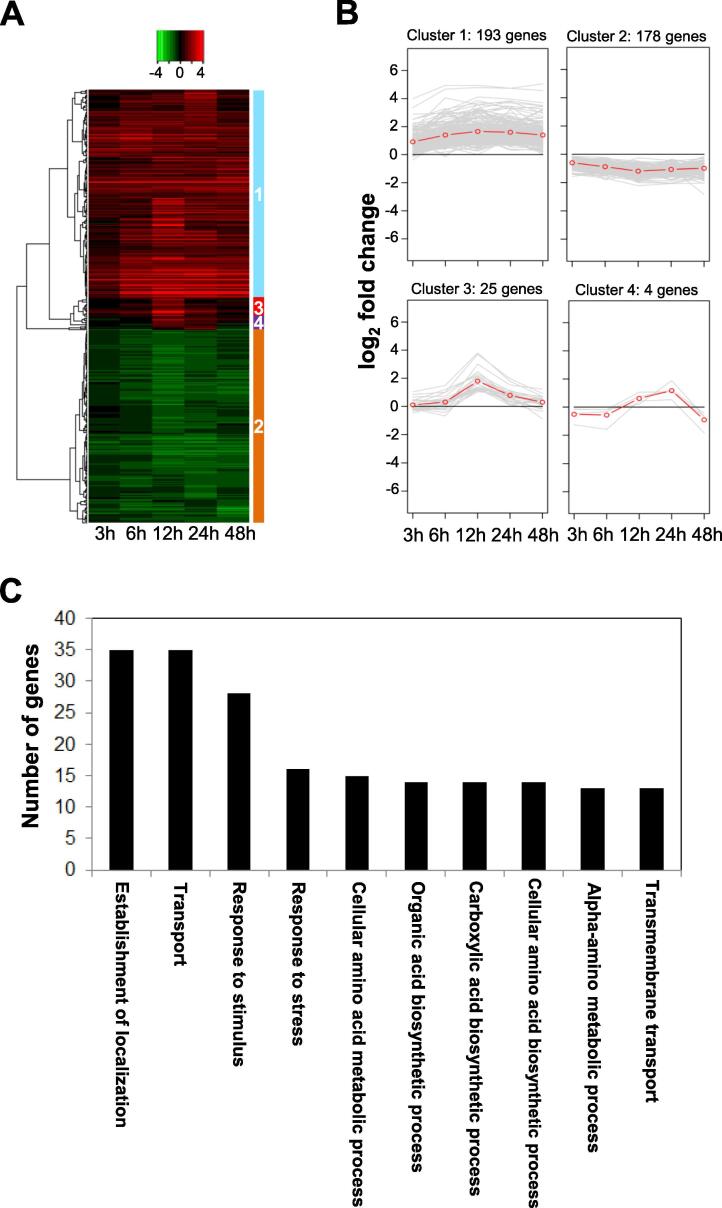
Expression analysis of *Vv* MO6 genes in infiltrated *A. thaliana* leaves. (A) A total of 811 differentially expressed genes (DEGs) of *Vv* MO6 were selected at 3, 6, 12, 24, and 48 h post-infiltration (hpi). Heat map analysis shows four independent clusters determined by unsupervised hierarchical analysis. Red and green colors indicate high and low expression levels, respectively. (B) Line plot analysis of the relative expression patterns of *Vv* MO6 genes at different time points. Most of the 400 clustered genes belonged to clusters 1 and 2. Red line indicates the mean expression of all genes at each time point. (C) Gene Ontology (GO) analysis of DEGs of *Vv* MO6. Genes in cluster 1 were enriched in the biological process GO category and were mainly involved in establishment of localization, transport, response to stimulus, and response to stress. (For interpretation of the references to colour in this figure legend, the reader is referred to the web version of this article.)

Compared with the 0 h time point*,* a total of 31, 128, 303, 219, and 130 genes were differentially expressed at 3, 6, 12, 24, and 48 hpi, respectively (Table 1) and overlapping DEGs were presented at indicated time points (Supplementary Fig. 2). Among these DEGs, the top ten up- and down-regulated genes were characterized (Supplementary Tables 2–6). Based on their characterization at each time point, the DEGs were classified into four independent clusters by unsupervised hierarchical clustering analysis (Fig. 2A). Of these, approximately 92.5% (371) genes were classified into clusters 1 and 2. According to line plot analyses, genes in clusters 1 and 2 were up-regulated and down-regulated, respectively, at all time points tested (Fig. 2B). Genes in cluster 3 genes were specifically up-regulated at 12 and 24 h, while those in cluster 4 showed mixed expression patterns (i.e., down-regulated at 3, 6 and 48 h but up-regulated at 12 and 24 h) (Fig. 2B). Eight genes belonged to cluster 1 were validated using real-time reverse transcription-PCR (qRT-PCR) and these gene were highly upregulated compared to 0 h control (Supplementary Fig. 3).

**Table 1:** Number of genes differentially expressed in *Vibrio vulnificus* MO6-24/O (*Vv* MO6) upon the infection of *Arabidopsis thaliana* plants at the indicated time points.

Comparison	Total number of DEGs^a^	Number of up-regulated DEGs	Number of down-regulated DEGs
0 h vs. 3 h	31	25	6
0 h vs. 6 h	128	80	48
0 h vs. 12 h	303	166	137
0 h vs. 24 h	219	128	91
0 h vs. 48 h	130	77	55

a DEGs, differentially expressed genes showing at least 2-fold difference in expression between treatments at the same time point.

Next, to predict the potential functions of genes in these four clusters, we performed GO enrichment analysis. Expression patterns of cluster 1 and 2 genes exhibited a contradictory mode, which hypothesized us that the patterns of biological process in between cluster 1 and 2 genes were highly different from each other. Genes in cluster 1 contributed to a total of 36 types of biological processes, including the establishment of localization, transport, response to stimulus and response to stress (Fig. 2C). Interestingly, although cluster 2 contained 178 genes, almost no biological process term was enriched among these genes (Supplementary Fig. 4).

Although we proved and verified that *Vv* MO6 causes disease symptom in *A. thaliana* in our previous study*,* the underlying molecular mechanism was not elucidated. The notion was somehow addressed in the current study based on the following results: 1) *Vv* MO6 and *A. thaliana* sequences were detected by RNA-seq; 2) a total of 811 DEGs were identified in *Vv* MO6 during the infection of *A. thaliana*; 3) the specific metabolic pathways of clustered genes were predicted and annotated by GO and KEGG analyses; and 4) the total transcripts of *Vv* MO6 were precisely detected and analyzed from *A. thaliana* plants. These results indicate that disease symptoms in *A. thaliana* were caused by *Vv* MO6, which is the first evidence in this field. Interestingly, unlike cluster 2 genes (down-regulated at all time points), cluster 1 genes (up-regulated at all time points) were involved in a variety of biological processes. This suggests that up-regulation of genes is required for inducing disease symptoms and modulating defense responses in *A. thaliana*. However, it may be still unclear how *Vv* MO6 regulates acidification, salinity, and CO_2_ levels to survive and maintain its virulence and pathogenicity in plant host [36], [37], [38], [39], [40], [41]. Therefore, if we analyzed genes involved in controlling the growth conditions of *Vv* MO6 or encoding its virulence and pathogenicity factors, the potential mechanisms of *Vv* MO6-induced disease in *A. thaliana* should be predictable.

### 3.3 Virulence and pathogenicity-associated *Vv* MO6 genes are up-regulated during the infection of *A. thaliana*

To determine how *Vv* MO6 survived and caused disease in *A. thaliana*, we screened the DEGs encoding virulence factors by tblastn (E-value ≤ 1e-10; identity ≥ 95%). The identified candidate genes were grouped into six categories: adherence, antiphagocytosis, chemotaxis and motility, iron uptake, secretion system, and toxin [42]. Of these, 14 candidate genes were characterized (Table 2). Three mannose-sensitive hemagglutinin (MSHA) biogenesis protein genes, *mshE*, *mshM*, and *mshN*, were up-regulated at 12 hpi. Antiphagocytosis-associated genes were broadly up-regulated at most time points. Among chemotaxis and motility genes, only the flagellar biosynthesis gene, *fliR*, was constantly up-regulated at all time points. The flagellar forming proteins (flagellins) are important pathogen-associated molecular pattern (PAMP) molecules for activating PAMP-triggered immunity (PTI) in plant and human [43]. In addition, the heme transport gene *hutR* was up-regulated at 6 hpi, and two RTX toxin-encoding gene *rtxD* and two type II secretion system component-encoding genes (*epsE* and *epsJ*) were slightly up-regulated in comparison with other genes (Table 2). These results suggest that the abovementioned genes play a key role in the virulence of *Vv* MO6, and function as modulators of plant immunity in *A. thaliana*. However, we do not conclude how toxins and secretion system of *Vv* MO6 regulate plant immune responses, resulting symptom development and increases of plant susceptibility.

**Table 2:** Virulence and pathogenicity factor related genes of *Vv* MO6 showing differential expression at indicated time points.

Category	Subcategory	Gene name	Gene description	Log_2_ (fold change)				
				3 h	6 h	12 h	24 h	48 h
Adherence	Mannose-sensitive hemagglutinin (MSHA type IV pilus)	*mshE*	MSHA biogenesis protein MshE			1.33		1.10
Adherence	Mannose-sensitive hemagglutinin (MSHA type IV pilus)	*mshM*	MSHA biogenesis protein MshM			1.30		
Adherence	Mannose-sensitive hemagglutinin (MSHA type IV pilus)	*mshN*	MSHA biogenesis protein MshN			1.54		
Antiphagocytosis	Capsular polysaccharide	*cpsC*	Polysaccharide export protein	2.35	1.61	1.58	1.29	1.02
Antiphagocytosis	Capsular polysaccharide	*cpsI*	Glycosyltransferase	2.00		1.40		
Antiphagocytosis	Capsular polysaccharide	*wbfV/wcvB*	UDP-glucose 6-dehydrogenase		1.41	1.38	1.19	1.42
Chemotaxis and motility	Flagella	*flhB*	Flagellar biosynthesis protein		1.13	1.34		1.29
Chemotaxis and motility	Flagella	*fliQ*	Flagellar biosynthesis protein			1.27	1.20	1.50
Chemotaxis and motility	Flagella	*fliR*	Flagellar biosynthesis protein		1.38	1.72	1.47	1.69
Chemotaxis and motility	Flagella	*flgL*	Flagellar hook-associated protein				1.06	1.06
Iron uptake	Heme receptors	*hutR*	Heme transport protein		1.30			
Toxin	RTX toxin	*rtxD*	RTX toxin transporter		1.01		1.16	1.61
Secretion system	EPS type II secretion system	*epsE*	Type II secretory pathway, ATPase EpsE			1.17		
Secretion system	EPS type II secretion system	*epsJ*	Type II secretory pathway, component EpsJ			1.10		

The *mshA* gene, which is highly identical to *mshE*, *mshM*, and *mshN,* encodes a type IV pilus subunit protein and is involved in the adhesion of *Vibrio parahaemolyticus* to its host [39], [40]. The expression level of *mshA* is reduced under aerobic conditions, resulting in decreased adhesion of *V. parahaemolyticus* to the host*.* This is because the decreased expression level of *mshA* inhibits the expression of *cpsA* and *cpsC*, which are involved in capsular polysaccharide (CPS) production and transportation, thus decreasing adhesion and biofilm formation in *V. parahaemolyticus* [41]. Our results indicated that up-regulation of *msh* genes and *cpsC* stimulated biofilm formation in *Vv* MO6 and enhanced its ability to adhere to *A. thaliana*, thus causing disease.

Previous evidence revealed that when *Vibrio* species infect animal hosts, the up-regulation of genes encoding toll-like receptors, RIG-like receptor, antioxidants and NF-kB signaling proteins was enhanced by the activation of innate immunity in the host cells [5], [6], [7]. These results suggest that *Vibrio*-mediated signals could be perceived by the membrane receptors of the host, and their signals may activate the downstream intracellular signaling pathways. Therefore, to understand *Vv* MO6–*A. thaliana* interactions, it is important to investigate the expression patterns of *A. thaliana* genes in response to *Vv* MO6 infection and to determine the role of certain receptor-mediated pathways and downstream defense responses.

### 3.4 *Vv* MO6 infection alters the expression levels of *A. thaliana* genes

To obtain molecular evidence supporting the notion that *Vv* MO6 modulates *A. thaliana* immunity, we analyzed the transcriptome of *A. thaliana* plants infiltrated with *Vv* MO6 or 10% PBS (negative control) at 3, 6, 12, 24, and 48 hpi. The relative expression levels of *A. thaliana* genes were quantified after *Vv* MO6 infiltration and compared with the negative control at the indicated time points. A total of 27,655 *A. thaliana* genes were used in this analysis, of which 21,662 were affected by *Vv* MO6 infiltration and annotated. In addition, trimmed reads corresponding to these genes were mapped on to the reference genome of *A. thaliana* (https://www.araport.org), with an average mapping rate of 92% across 20 samples. The correlation coefficients of replicates were very high (range: 0.98–0.99), indicating that no variation among replicates (Supplementary Fig. 5).

**Fig. 3:**
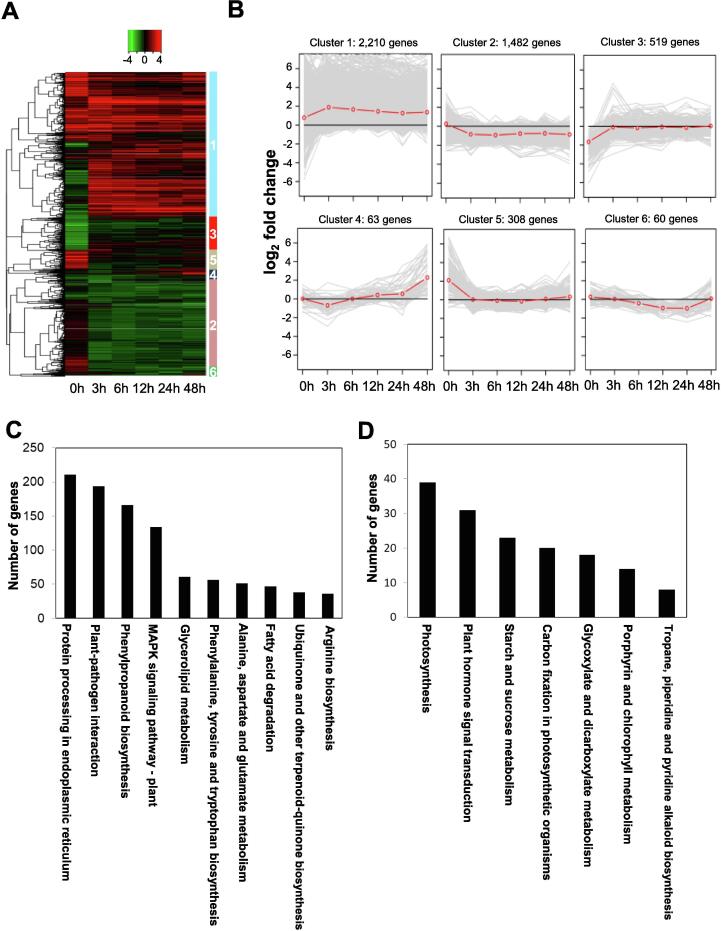
*Vv* MO6 modulates the *A. thaliana* transcriptome upon infiltration. (A) A total of 7,709 DEGs of *A. thaliana* were selected at 3, 6, 12, 24, and 48 hpi. Heat map analysis shows six independent clusters determined by unsupervised hierarchical analysis. Red and green colors indicate high and low expression levels, respectively. (B) Line plot analysis of the relative expression patterns of *A. thaliana* genes at different time points. Of the 4,642 clustered genes, approximately 80% genes belonged to clusters 1 and 2. Red line indicates the mean expression level of all genes at each time point. (C and D) GO and KEGG analyses of DEGs of *A. thaliana* in cluster 1 (C) and cluster 2 (D). (For interpretation of the references to colour in this figure legend, the reader is referred to the web version of this article.)

Comparison between *Vv* MO6-infiltrated and 10% PBS-infiltrated *A. thaliana* plants revealed 2,097, 1,839, 1,220, 1,170, and 1,383 DEGs at 3, 6, 12, 24, and 48 hpi, respectively (Table 3), and overlapping DEGs were presented at indicated time points (Supplementary Fig. 6). Among these, the identity, description and expression fold-change of the top 20 up- and down-regulated genes are listed in Supplementary Tables 7–11. These DEGs were classified into six independent groups by unsupervised hierarchical clustering analysis (Fig. 3A). Approximately 80% (3,692) of these genes were grouped in clusters 1 and 2. Line plot analysis revealed that cluster 1 genes were up-regulated at each time point after *Vv* MO6 infiltration, whereas cluster 2 genes were down-regulated post infiltration (Fig. 3B). Cluster 3 and 5 genes were not affected by *Vv* MO6 infiltration at any of these time points; cluster 4 genes were down-regulated at 3 hpi and up-regulated at 12–48 hpi; and cluster 6 genes were down-regulated at 6–24 hpi (Fig. 3B). Seven genes belonged to cluster 1 were validated using qRT-PCR and these gene were highly upregulated in comparison with controls (Supplementary Fig. 7).

**Table 3:** Number of *A. thaliana* genes differentially expressed between *Vv* MO6- and 10% PBS (control)-infiltrated plants at the indicated time points.

Time point	Total number of DEGs^a^	Number of up-regulated DEGs	Number of down-regulated DEGs
3 h	2,097	1,506	591
6 h	1,839	1,092	747
12 h	1,220	816	404
24 h	1,170	769	401
48 h	1,383	874	509

a DEGs, differentially expressed genes showing at least 2-fold difference in expression between treatments at the same time point.

The results of GO and KEGG analyses showed drastic differences between cluster 1 and 2 genes. Cluster 1 genes were broadly involved in protein processing in endoplasmic reticulum, plant-pathogen interaction, phenylpropanoid biosynthesis, and mitogen-activated protein (MAP) kinase signaling pathway (Fig. 3C). In contrast to cluster 1 genes, cluster 2 genes were predominantly related with photosynthesis and plant hormone signaling transduction (Fig. 3D). In addition, specific metabolic pathways were analyzed and summarized in Supplementary Fig. 8).

### 3.5 *A. thaliana* PRRs activate downstream signaling pathways upon the perception of *Vv* MO6

**Fig. 4:**
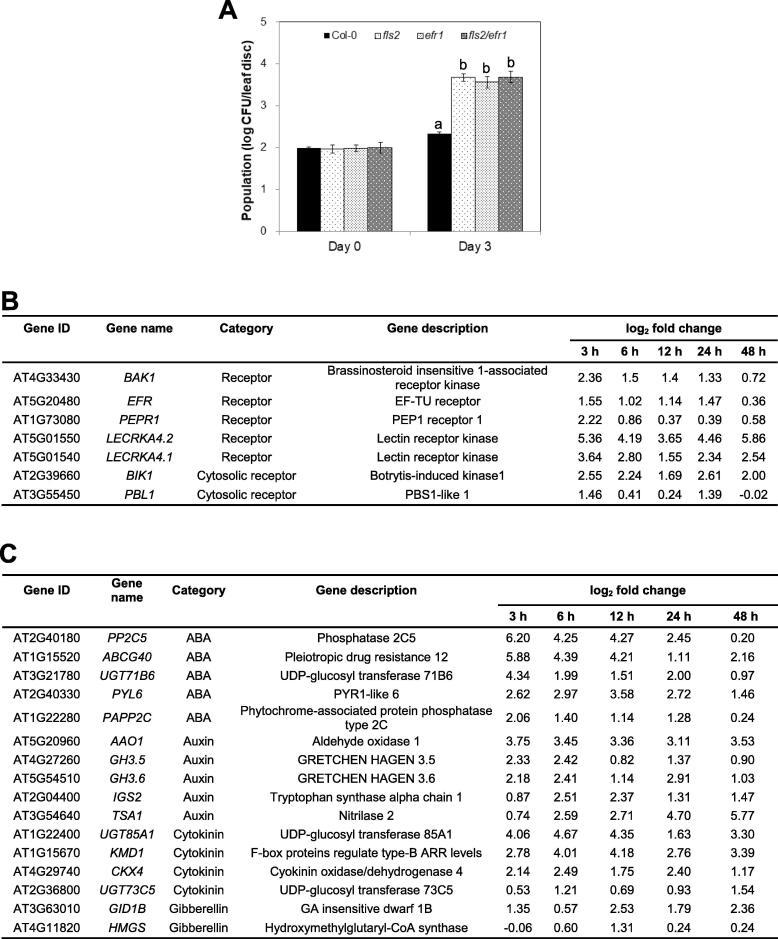
Pattern recognized receptors (PRRs) of *A. thaliana* perceive *Vv* MO6 signals and activate downstream pathways. (A) Population density of *Vv* MO6 in three mutant lines at 3 dpi. Disruption of *PRR* genes resulted in approximately 15-fold increase in population density. Different letters indicate significant differences (*P* = 0.05) between genotypes. (B) Seven *A. thaliana* DEGs encoding *PRR* genes post-infiltration with *Vv* MO6. (C) Characterization of 10 DEGs in *A. thaliana* involved in reactive oxygen species (ROS) burst and mitogen-activated protein (MAP) kinase cascade.

To investigate whether and how *A. thaliana* plants perceive *Vv* MO6, the abundance of *Vv* MO6 was quantified in *A. thaliana* Col-0 (wild type) plants and knockout mutants of genes that perceive the PAMPs or MAMPs such as flagellin and elongation factor [43]. Bacterial colonies were counted in Col-0 plants and *flagellin sensing 2* (*fls2*) and *elongation factor 2 related 1* (*efr1*) knockout mutants at 3 dpi. The *fls2* and *efr1* mutants showed approximately 15-fold higher abundance of *Vv* MO6 than Col-0 plants (Fig. 4A), suggesting that *FLS2* and *EFR1* are required for recognizing the molecular patterns of *Vv* MO6, and the recognized receptors play a pivotal role in activating intracellular downstream signaling pathways.

In agreement with genetic analysis of PRRs, transcriptome analysis revealed that *EFR*, *PEP1 receptor 1* (*PEPR1*) and *Brassinosteroid insensitive 1-associated receptor kinase* (*BAK1*) were up-regulated in *Vv* MO6-infiltrated plants compared with control plants (Fig. 4B). Additionally, mRNA levels of *Lectin receptor kinase 4.1* (*LECRKA 4.1*) and *LECRKA 4.2*, which encode other type of receptors, were significantly higher in *Vv* MO6-infiltrated plants than in control plants (Fig. 4B). It is known that signals activated by PRRs are transferred to the cytoplasmic receptors in plant innate immunity [44]. Therefore, we monitored the transcript levels of two well-known cytosolic receptor-encoding genes, *Botrytis-induced kinase 1* (*BIK1*) and *PBS1-like 1* (*PBL1*). The results showed that *BIK1* was up-regulated at all tested time points, and *PBL1* was significantly up-regulated at 3 and 24 hpi (Fig. 4B).

Reactive oxygen species (ROS) burst and the induction of mitogen-activated protein (MAP) kinase cascade are necessary for activating plant immunity [45]. Consistent with this observation, we found that the *Respiratory burst oxidase homolog D* (*RBOHD*) gene was slightly up-regulated at 3 hpi (Fig. 4C). In addition, three *MAP kinase kinase kinase* (*MAPKKK*) genes, two *MAP kinase kinase* (*MAPKK, MKK*) genes and four *MAP kinase* (*MAPK, MPK*) genes were identified as DEGs (Fig. 4C). Among these genes, *MAPKKK15*, *MAPKKK19* and *MPK11* were highly up-regulated at all time points (Fig. 4C).

Plant defense machinery is activated by plant pathogens [43]. Similarly, our results suggest that *Vv* MO6 signals are recognized by the common PRRs (e.g., EFR1) and subsequently interact with co-receptors (e.g., BAK1). The activation of *Vv* MO6 signals by PRRs and co-receptors stimulates cytosolic receptors (such as BIK1 and PBL1), leading to ROS burst and MAPK cascade induction. The perception of PAMPs/MAMPs by PRRs activates defense responses, referred to as PTI [46]. For example, the perception of PAMPs by PRRs triggers the production of hydrogen peroxide (H_2_O_2_) mainly by the action of RBOHD [47]. In addition, the perception of PAMPs by PRRs leads to the activation of MAP kinases. Previously, flg22 treatment revealed that at least six MPKs and MPK3/MPK6 are dependent on the upstream MKK4/MKK5 proteins [48]. To overcome PTI, pathogens inject virulence effectors into the host cells, resulting in disease. This phenomenon is referred to as effector-triggered susceptibility (ETS) [49]. Subsequently, host *resistance* (*R*) genes recognize the effectors and terminate pathogen growth, resulting in robust immunity, termed as effector-triggered immunity (ETI) [50]. This information suggests that *Vv* MO6 potentially secretes effector proteins into the *A. thaliana* cells to shut down PTI, and then certain *R* genes directly or indirectly recognize *Vv* MO6 effector proteins. It is noteworthy that *V. vulnificus*, a marine bacterium, can manipulate the immune system of *A. thaliana*, a land plant.

### 3.6 *Vv* MO6 regulates hormone-related genes in *A. thaliana*

**Fig. 5:**
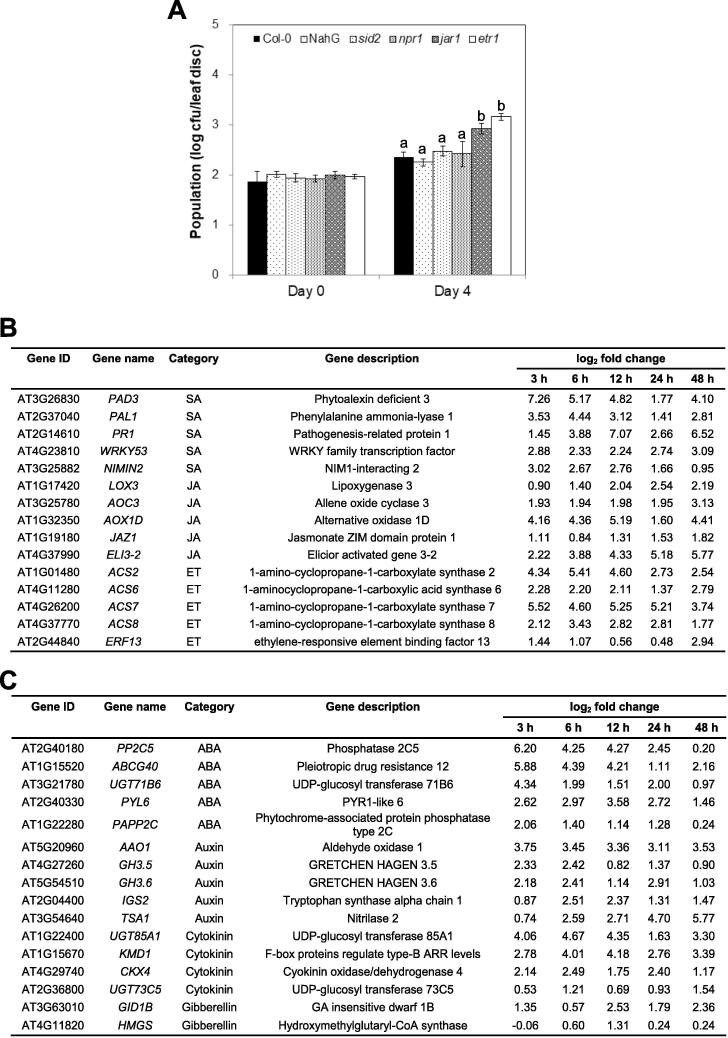
*Vv* MO6 modulates phytohormone-mediated pathways in *A. thaliana*. (A) Quantification of the population density of *Vv* MO6 in wild-type (Col-0) and mutant plants. several mutant lines including SA signaling pathways NahG transgenic, SA-deficient mutant (*sid2*), SA-insensitive mutant (*npr1*), JA response mutant (*jar1*) and *Ethylene Receptor 1* mutant (*etr1*). The population density of *Vv* MO6 was 12- and 13.5-fold higher in *jar1* and *etr1* mutants, respectively, than in Col-0 plants. Different letters indicate significant differences (*P* = 0.05) between genotypes. (B) Top five DEGs involved in defense hormone related pathways. Genes involved in salicylic acid (SA)-, jasmonic acid (JA)-, and ethylene (ET)-dependent pathways are shown. (C) Selected DEGs involved in abscisic acid (ABA), auxin, cytokinin (CK), and gibberellin (GA) related pathways.

Salicylic acid (SA), jasmonic acid (JA) and ethylene (ET) function as major defense related hormones in plants [51]. To verify the potential roles of these hormones in defense against *Vv* MO6, we used a genetic approach. The population density of *Vv* MO6 was quantified in *Vv* MO6-infiltrated Col-0 (wild type) plants, and several mutant lines including SA-deficient NahG transgenic and SA-deficient mutant (*sid2)*, SA-insensitive mutant (*nonexpressor of pathogenisis-related gene 1*; *npr1*), JA response mutant (*jar1*), and ethylene insensitive mutant (*ethylene receptor 1*; *etr1*). The results showed no difference in the population density of *Vv* MO6 between Col-0 and SA deficient or insensitive lines at 4 dpi (Fig. 5A). However, the population density of *Vv* MO6 was 12- and 13.5-fold higher in *jar1* and *etr1* mutants, respectively, than in Col-0 plants (Fig. 5A).

Five representative genes of the SA, JA, and ET signaling pathways are shown in Fig. 5B. The expression levels of genes such as *phytoalexin deficient 3* (*PAD3*), *pathogenesis-related protein 1* (*PR1*), *alternative oxidase 1D* (*AOX1D*), *elicitor activated gene 3*–*2* (*ELI3-2*), *1-amino-cyclopropane-1-carboxylate synthase 2* (*ACS2*) and *1-amino-cyclopropane-1-carboxylate synthase 6* (*ACS6*) were relatively higher than those of other genes at all time points (Fig. 5B). In addition to the defense hormone related genes, the ABA, auxin, CK, and GA related genes were selected in Fig. 4C. The up-regulation of GA pathway-associated genes was relatively weaker than that of other hormone pathway-associated genes. Additionally, genes such as *ATP-binding cassette G40* (*ABCG40*), *PYR1-like 6* (*PYL6*), *aldehyde oxidase 1* (*AAO1*), *GRETCHEN HAGEN 3.6* (*GH3.6*), *UDP-glucosyl transferase 85A1* (*UGT85A1*), *kiss me deadly 2* (*KMD2*) and *cytokinin oxidase/dehydrogenase 4* (*CKX4*) were differentially up-regulated at all time points (Fig. 5C).

Although RNA-seq analysis showed that SA, JA, and ET pathway related genes were highly up-regulated in *Vv* MO6-infiltrated plants, our genetic analyses indicated plant defense against *Vv* MO6 relied only on JA and ET pathways. This observation is consistent with our previous study [8]. The difference between the results of RNA-seq analysis and genetic analysis may be explained based on two hypotheses: 1) the SA deficient or insensitive mutants used in this study did not represent the SA related genes involved in the response to *V. vulnificus*; 2) *V. vulnificus* modulates the transcript levels of SA, JA and ET genes, but only JA and ET related genes regulate plant immunity against *V. vulnificus* at the post-transcriptional and post-translational levels. Further research is needed to test these hypotheses. Nonetheless, it is intriguing that pathogen can regulate genes related to plant growth and development hormones such as ABA, auxin, CK and GA. These results suggest that sophisticated phytohormone signaling pathways interact with each other to defend the plant against *V. vulnificus.*

## 4 Conclusions

**Fig. 6:**
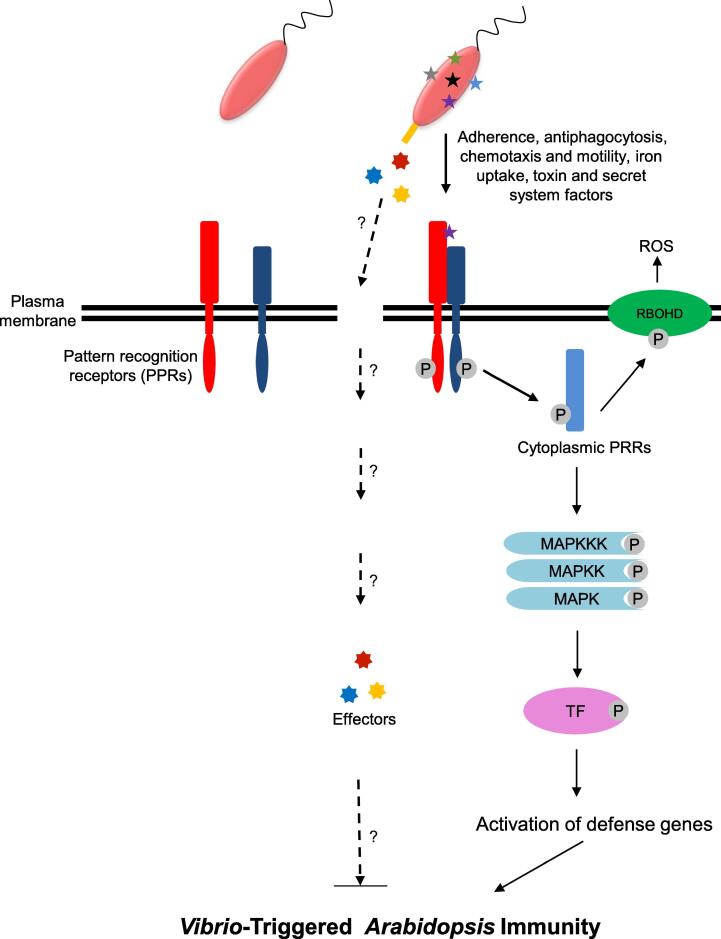
Schematic representation of the proposed *A. thaliana* –*Vv* MO6 interaction model.

In the current study, *Vv* MO6 clearly modulates its own transcripts and its plant host simultaneously under favorable conditions. To the best of our knowledge, this is the first time that the transcriptome of a marine bacterial pathogen is conclusively analyzed in a plant host. As our intriguing findings are summarized in Fig. 6, *Vv* MO6, approximately 92.5% (371) genes were up- or down-regulated during its interaction with *A. thaliana*. More intriguingly, the expression of virulence and pathogenicity-associated genes in bacterium may be required for inducing disease and modulating plant immune responses. Thus, our results shed light on the cross-talking between a human marine pathogen and a plant host. Our data provide a new strategy to identify important factors of animal and human pathogens by using plants as alternative hosts. Collectively, our results reveal a unique scenario in which a human pathogenic bacterium and a plant communicate simultaneously within the same space under artificial and favorable conditions.

## CRediT authorship contribution statement

**Yong-Soon Park:** Conceptualization, Methodology, Formal analysis, Supervision, Funding acquisition, wrote original paper. **Jong-Seok Park:** Conceptualization, Resources, Funding acquisition. **Soohyun Lee:** Methodology. **Sung-Hee Jung:** Methodology. **Seon-Kyu Kim:** Conceptualization, Methodology. **Choong-Min Ryu:** Conceptualization, Supervision, Project administration, Funding acquisition.

## Declaration of Competing Interest

The authors declare that they have no known competing financial interests or personal relationships that could have appeared to influence the work reported in this paper.

## Appendix A Supplementary data

Supplementary Fig. 1 Scatterplot matrices of *Vibrio vulnificus* MO6-24/O (*Vv* MO6)-infiltrated *A. thaliana* samples. The correlation coefficients of samples were very high (0.92–0.97), indicating no variation among samples.

Supplementary Fig. 2 Overlapping DEGs of *Vv* MO6 at indicated time points.

Supplementary Fig. Fig. 3 Validation of *Vv* MO6 expression levels using qRT-PCR.

Supplementary Fig. Fig. 4 Gene Ontology (GO) analysis of cluster 2 of *Vv* MO6.

Supplementary Fig. 5 Scatterplot matrices of *A. thaliana* samples. The correlation coefficients of all samples were very high (0.98–0.99), indicating no variation among samples.

Supplementary Fig. 6 Overlapping DEGs of *A. thaliana* at indicated time points.

Supplementary Fig. 7 Validation of *A. thaliana* expression levels using qRT-PCR.

Supplementary Fig. 8 Some metabolic pathway analyses in cluster 1 and 2 of *A. thaliana*.

Supplementary Table 1 Primer sequences used in this study.

Supplementary Tables 2–6 Supplementary Table 2. Top 10 differentially expressed genes (DEGs) of Vibrio vulnificus MO6-24/O (*Vv* MO6) at 3 h post-infiltration (hpi) of *A. thaliana* leaves. Supplementary Table 3. Top 10 DEGs of *Vv* MO6 at 6 hpi of *A. thaliana* leaves. Supplementary Table 4. Top 10 DEGs of *Vv* MO6 at 12 hpi of *A. thaliana* leaves. Supplementary Table 5. Top 10 DEGs of *Vv* MO6 at 24 hpi of *A. thaliana* leaves. Supplementary Table 6. Top 10 DEGs of *Vv* MO6 at 48 hpi of *A. thaliana* leaves.

Supplementary Tables 7–11 Supplementary Table 7. Top 20 DEGs of *A. thaliana* at 3 hpi with *Vv* MO6. Supplementary Table 8. Top 20 DEGs of *A. thaliana* at 6 hpi with *Vv* MO6. Supplementary Table 9. Top 20 DEGs of *A. thaliana* at 12 hpi with *Vv* MO6. Supplementary Table 10. Top 20 DEGs of A. thaliana at 24 hpi with *Vv* MO6. Supplementary Table 11. Top 20 DEGs of *A. thaliana* at 48 hpi with *Vv* MO6.
