# Supplementary figures and images for: Simultaneous profiling of Arabidopsis thaliana and Vibrio vulnificus MO6-24/O transcriptomes by dual RNA-seq analysis
Source: Comput Struct Biotechnol J. 2021 Apr 8;19:2084–96. doi: 10.1016/j.csbj.2021.04.008 (PMC8085779; doi:10.1016/j.csbj.2021.04.008)

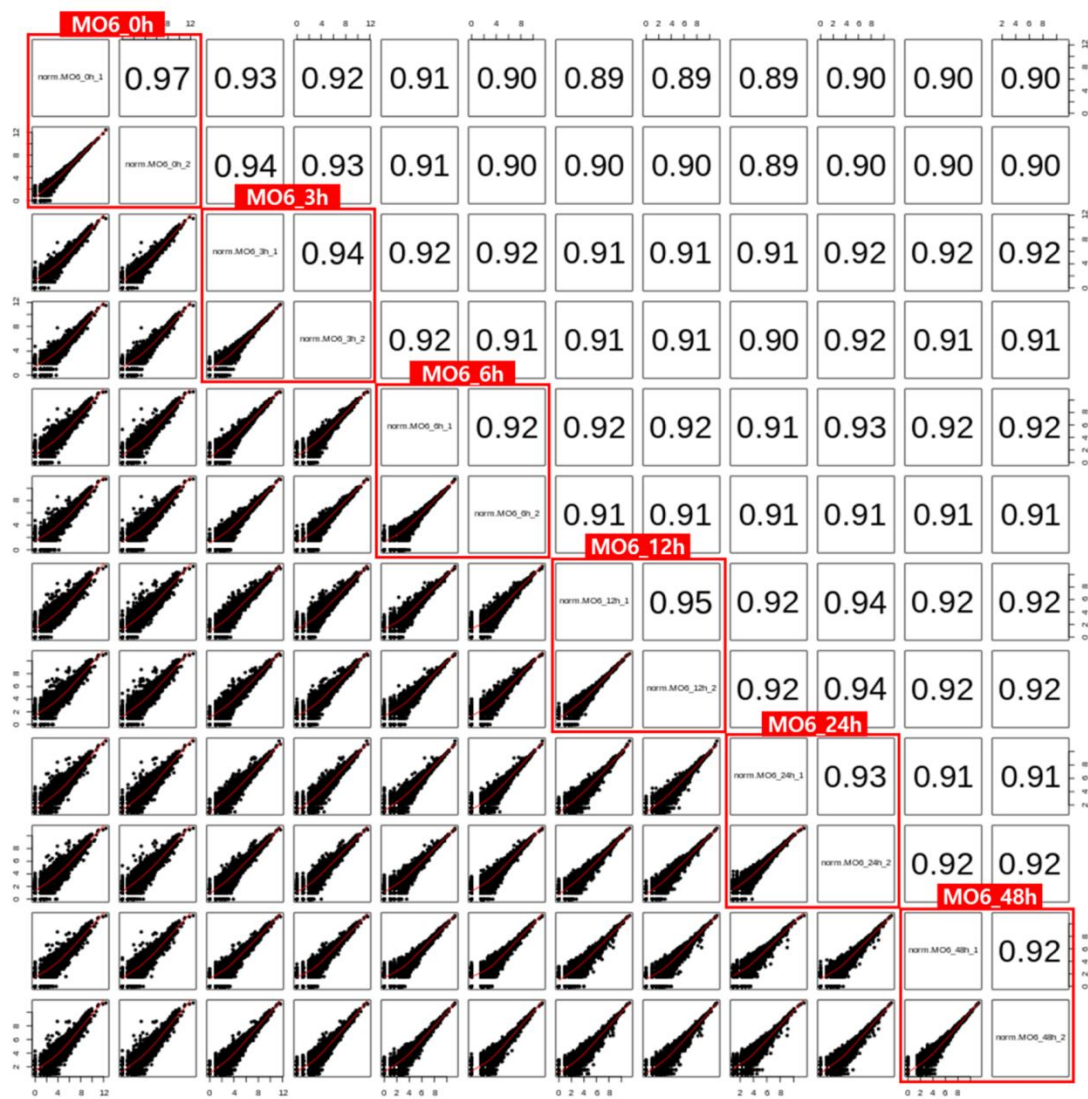

Supplement: Supplementary Fig. 1 — Scatterplot matrices of Vibrio vulnificus MO6-24/O (Vv MO6)-infiltrated A. thaliana samples. The correlation coefficients of samples were very high (0.92–0.97), indicating no variation among samples. [file mmc1.pdf]

Up-regulated DEGs

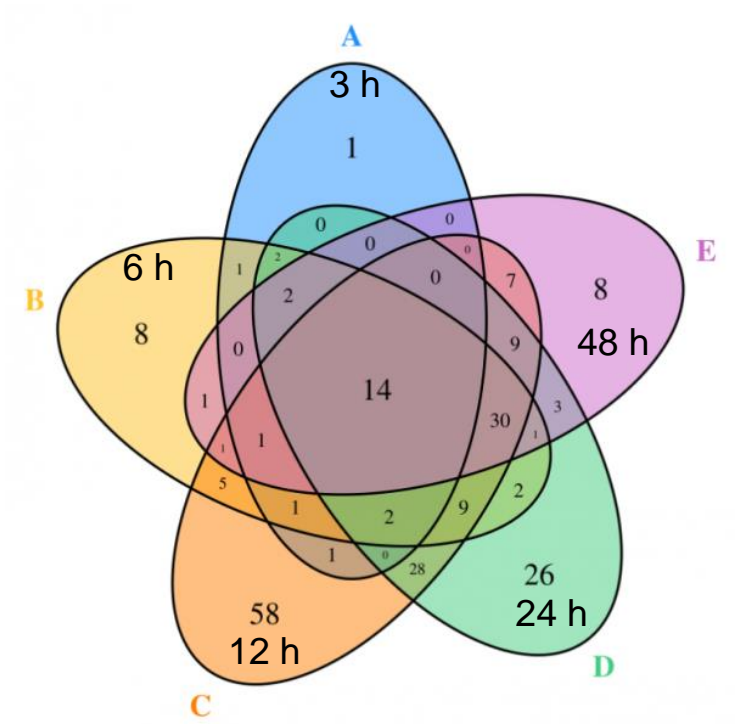

Down-regulated DEGs

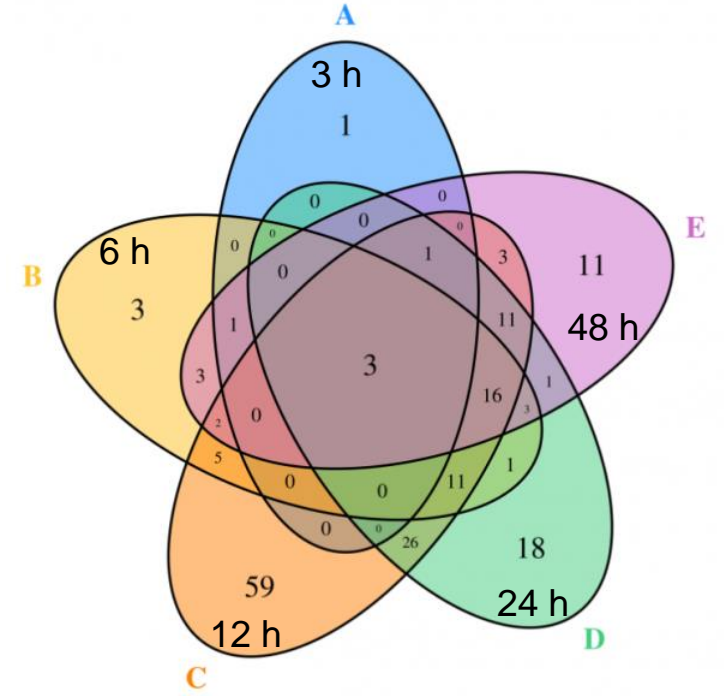

Supplement: Supplementary Fig. 2 — Overlapping DEGs of Vv MO6 at indicated time points. [file mmc2.pdf]

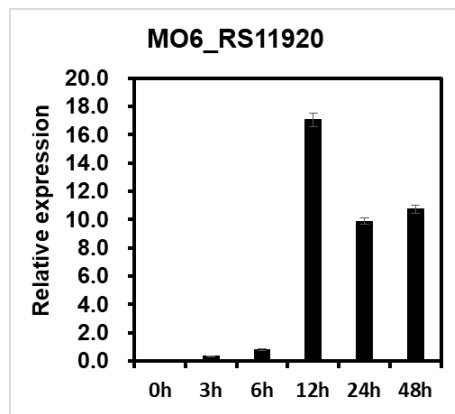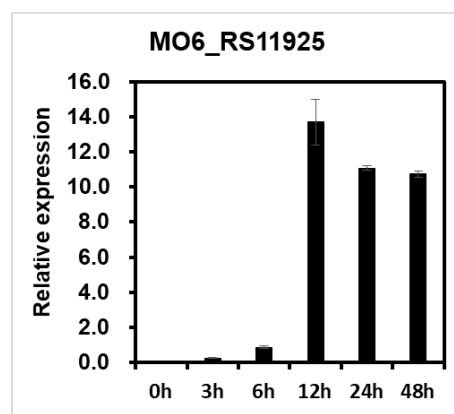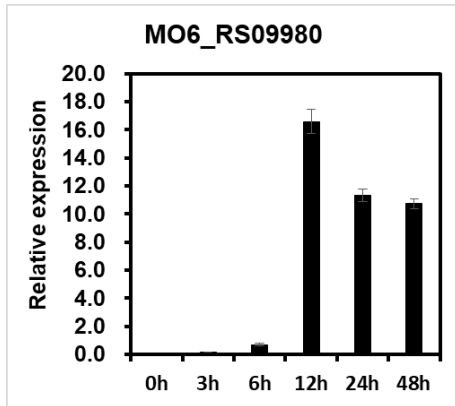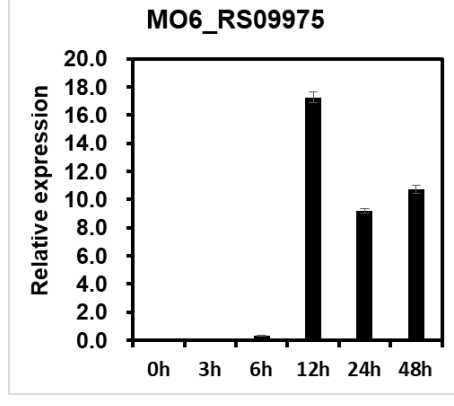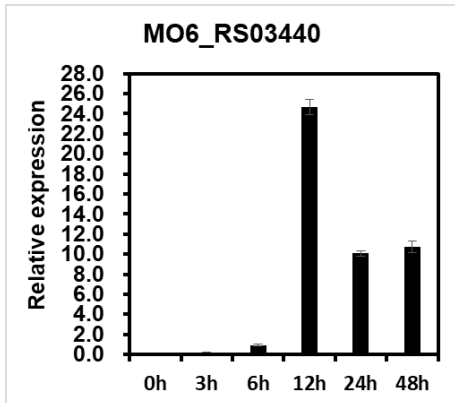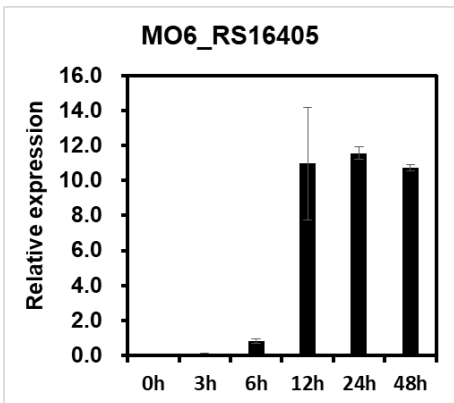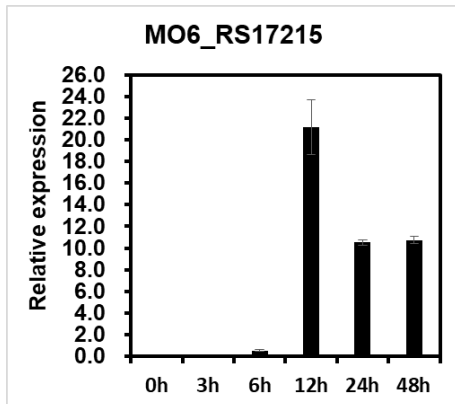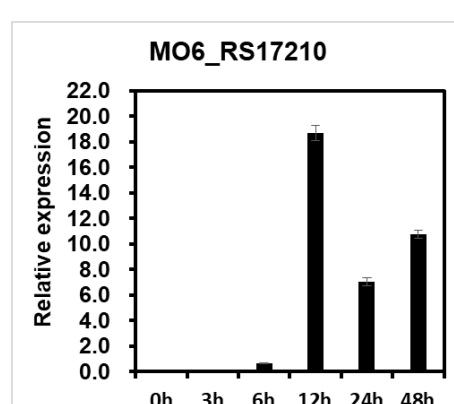

Supplement: Supplementary Fig. Fig. 3 — Validation of Vv MO6 expression levels using qRT-PCR. [file mmc3.pdf]

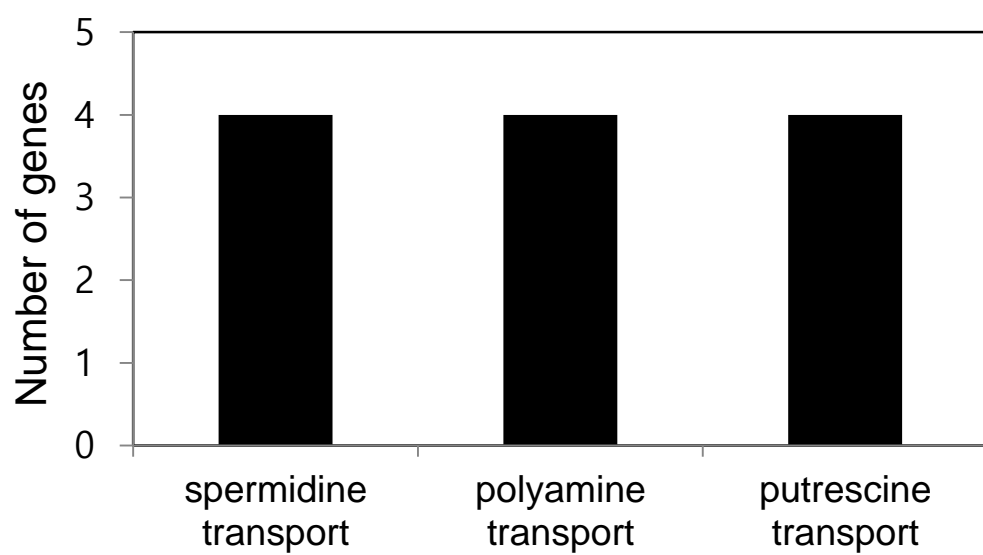

Supplement: Supplementary Fig. Fig. 4 — Gene Ontology (GO) analysis of cluster 2 of Vv MO6. [file mmc4.pdf]

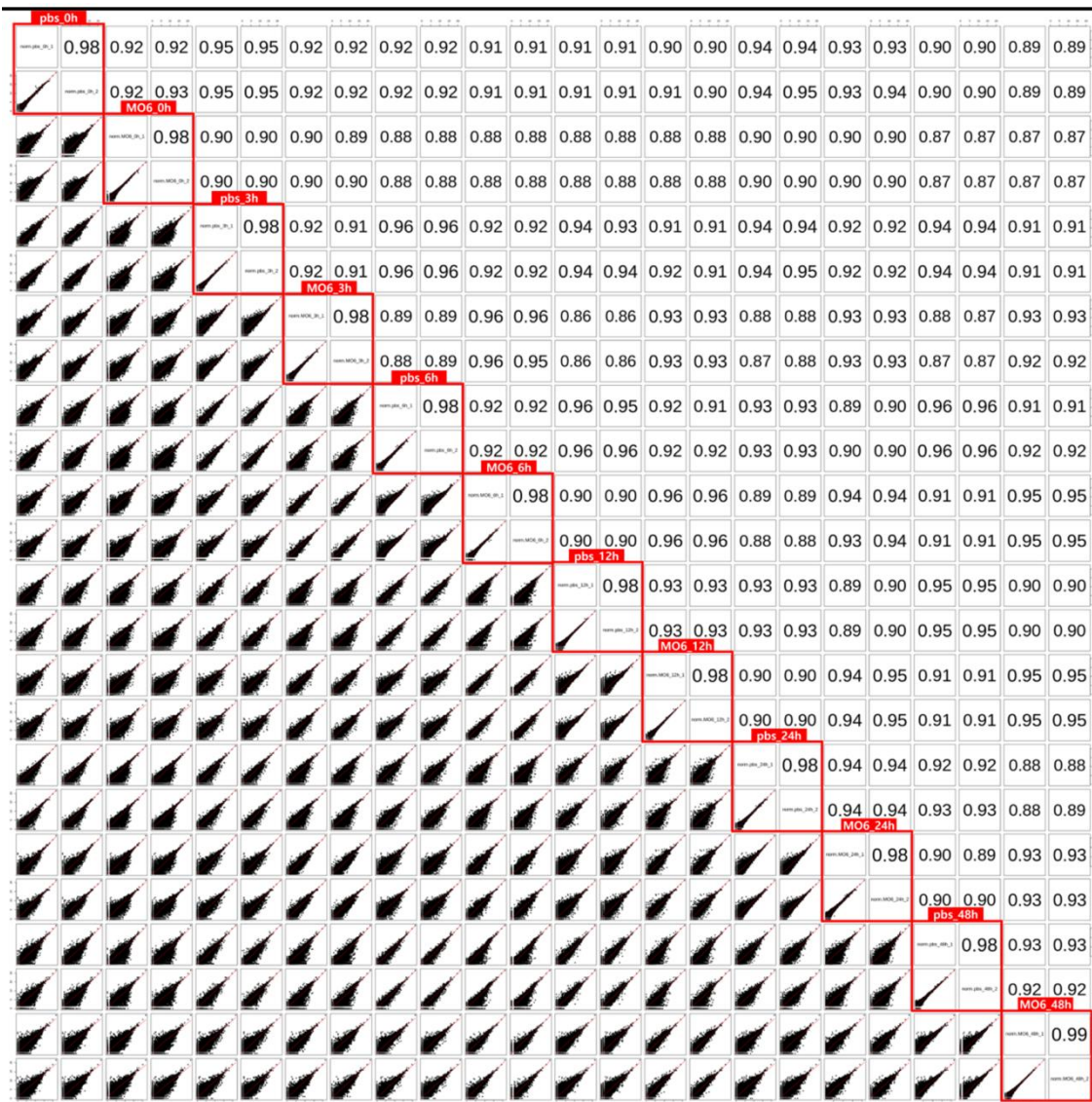

Supplement: Supplementary Fig. 5 — Scatterplot matrices of A. thaliana samples. The correlation coefficients of all samples were very high (0.98–0.99), indicating no variation among samples. [file mmc5.pdf]

## Up-regulated DEGs

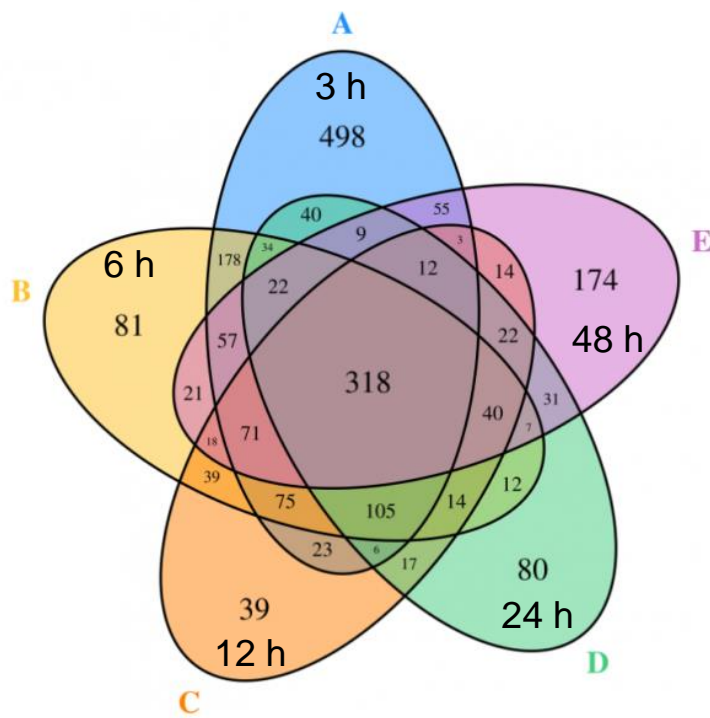

## Down-regulated DEGs

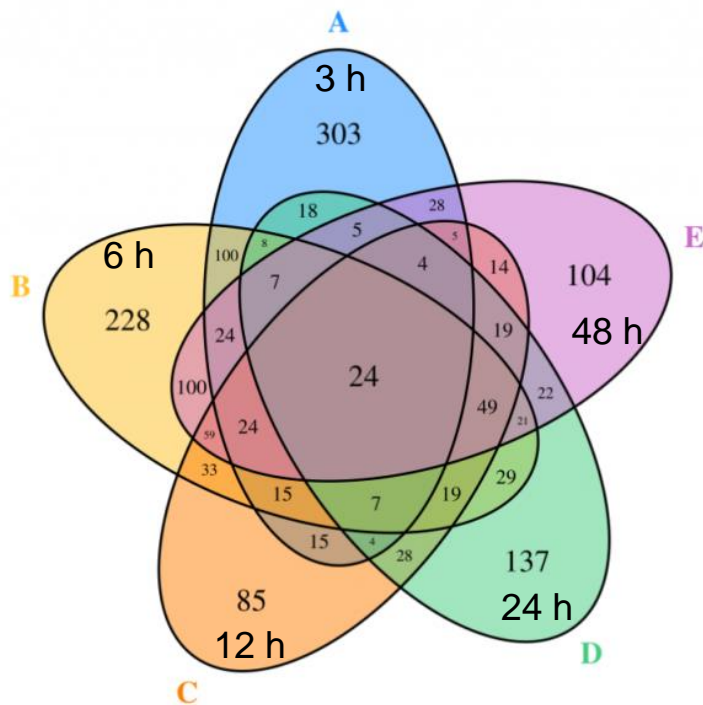

Supplement: Supplementary Fig. 6 — Overlapping DEGs of A. thaliana at indicated time points. [file mmc6.pdf]

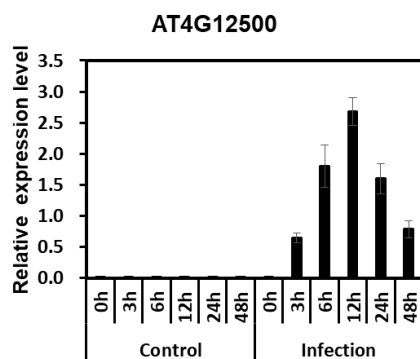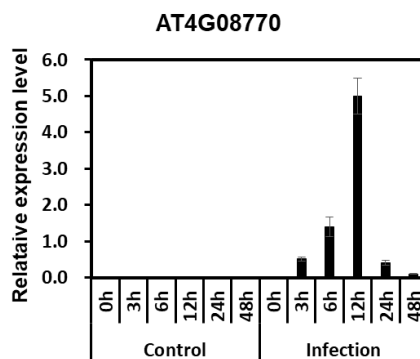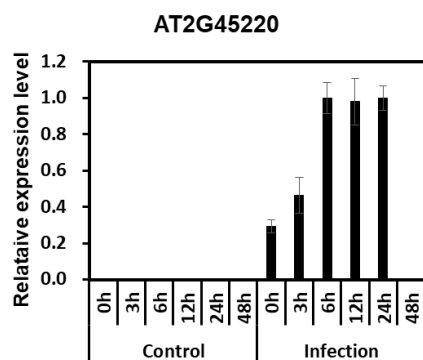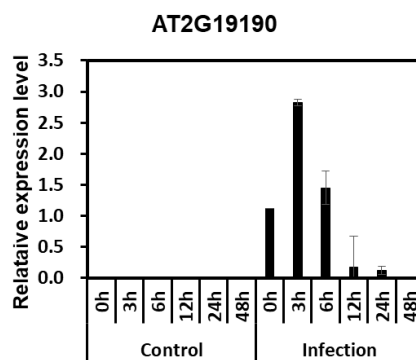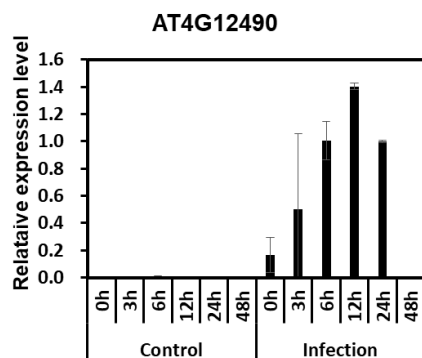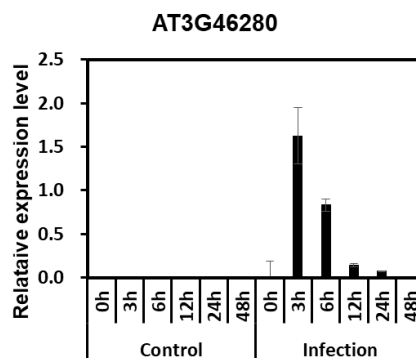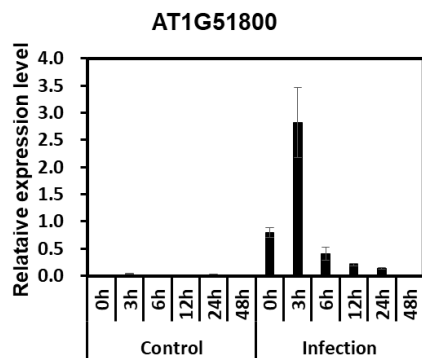

Supplement: Supplementary Fig. 7 — Validation of A. thaliana expression levels using qRT-PCR. [file mmc7.pdf]

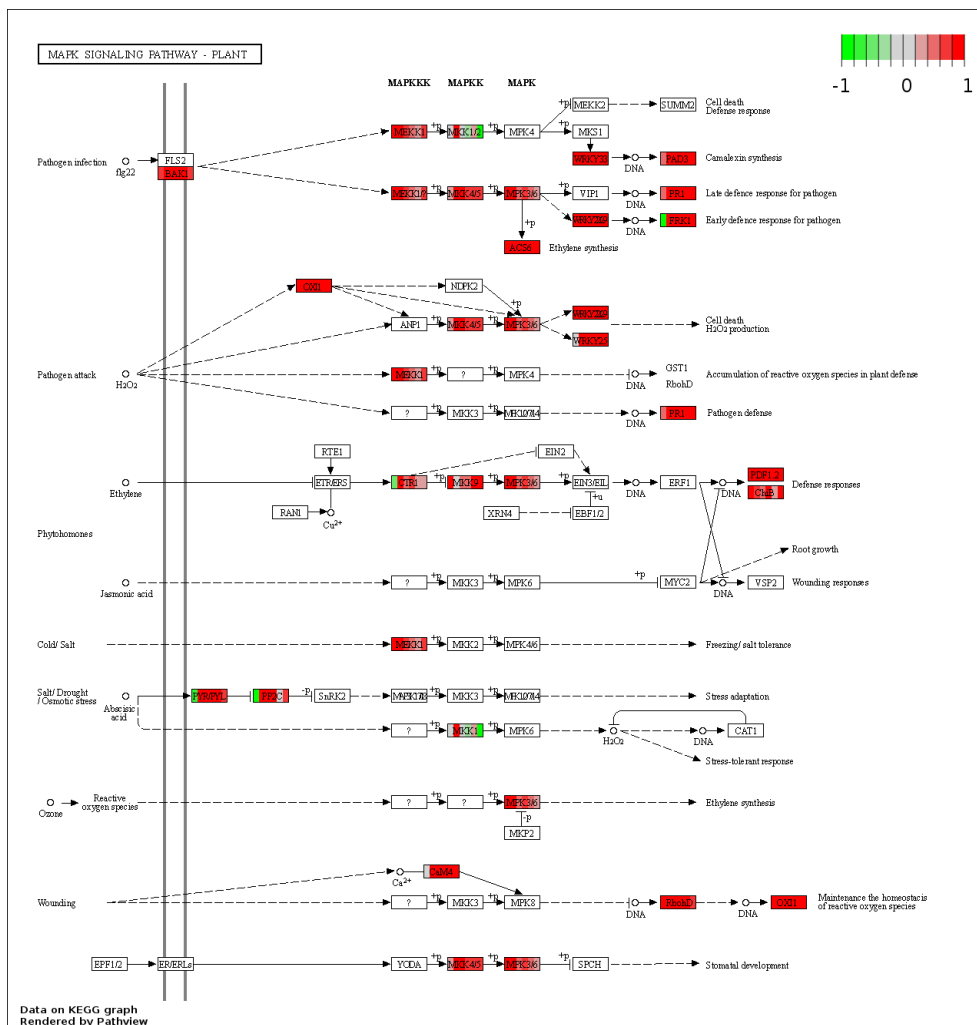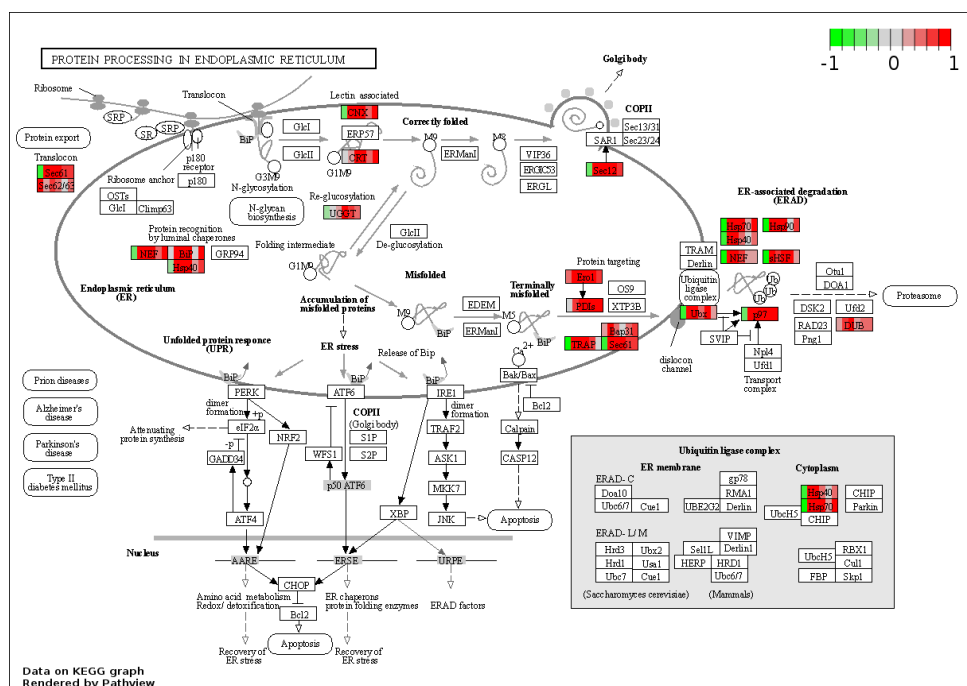



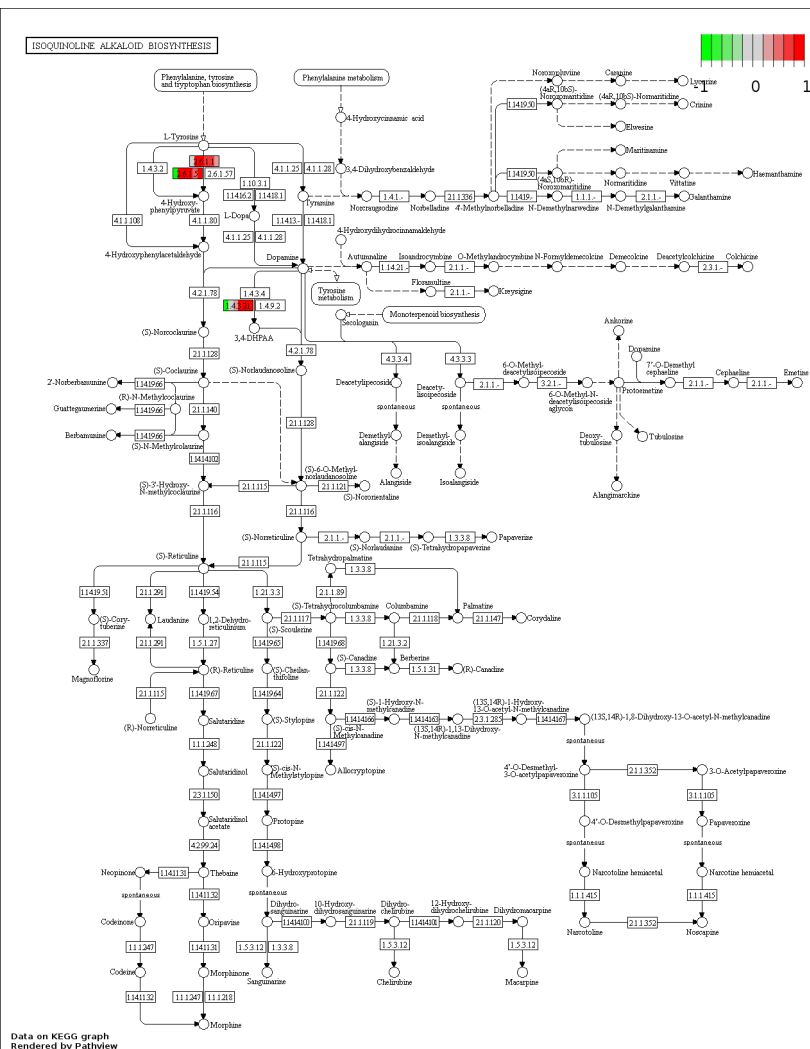

Supplement: Supplementary Fig. 8 — Some metabolic pathway analyses in cluster 1 and 2 of A. thaliana. [file mmc8.pdf]
